# Comparison of Intraneural Facilitation^TM^ Therapy and Exercise on Patients with Type 2 Diabetes: A Single-Blind Randomized Trial

**DOI:** 10.3390/biomedicines13122968

**Published:** 2025-12-03

**Authors:** Kyan Sahba, Christopher G. Wilson, Evelen Gonzales, Jamie Hankins, Hailey Jahromi, Mark Ghamsary, Mark Bussell

**Affiliations:** 1 Neuropathic Therapy Center, Loma Linda University Health, Loma Linda, CA 92354, USAmbussell@llu.edu (M.B.); 2 Lawrence D Longo MD Center for Perinatal Biology, Loma Linda University, Loma Linda, CA 92354, USA; 3 School of Nursing, Northern Kentucky University, Highland Heights, KY 41099, USA; 4 College of Osteopathic Medicine, Western University of Health Sciences, Redlands, CA 92373, USA; 5 Department of Epidemiology and Biostatistics, Loma Linda University, Loma Linda, CA 92354, USA

**Keywords:** diabetic peripheral neuropathy, intraneural facilitation therapy, physical therapy, neuropathic pain, type 2 diabetes, pain quality assessment scale, randomized control trial

## Abstract

**Background:** Diabetic peripheral neuropathy (DPN) is a prevalent complication of type 2 diabetes (T2D), associated with microvascular dysfunction and significant morbidity. Exercise is a cornerstone of diabetes care and has demonstrated benefits for neuropathic pain, whereas Intraneural Facilitation^TM^ (INF^®^) therapy is a manual technique designed to enhance intraneural perfusion. This study compared the effects of INF^®^ therapy and exercise on neuropathic pain qualities in adults with DPN. **Methods:** In this single-blinded randomized controlled trial, 38 adults with T2D and moderate to severe DPN were randomized to INF^®^ therapy (*n* = 20) or standardized exercise (*n* = 18). Participants completed nine 60-min sessions over a period of six weeks. Neuropathic pain qualities were assessed using the Pain Quality Assessment Scale (PQAS) at baseline and post-treatment. Paired *t* tests, independent *t* tests, and linear mixed models adjusted for age and body-mass index (BMI) evaluated within- and between-group changes. **Results:** Both treatment groups demonstrated significant reductions in total PQAS scores (*p* = 0.001). INF^®^ therapy produced improvements across paroxysmal, superficial, and deep pain domains, with reductions in descriptors such as shooting, sharp, electrical, numb, and unpleasant pain. Exercise led to selective improvements, including sharp, electrical, numb, sensitive, and unpleasant sensations associated with pain. Between-group analyses and mixed-effects models revealed no significant differences after adjusting for confounding factors. **Conclusions:** Both INF^®^ therapy and exercise improved neuropathic pain qualities in adults with DPN. INF^®^ therapy demonstrated broader within-group effects, suggesting its potential as a passive adjunct or alternative for patients unable to tolerate active exercise.

## 1. Introduction

Diabetic peripheral neuropathy (DPN) is one of the most prevalent microvascular complications of both type 1 (T1D) and type 2 diabetes (T2D) [1,2]. DPN affects up to 50% of adults with diabetes during their lifetime and is characterized by pain, paresthesia, progressive sensory loss, and distal symmetric polyneuropathy [3]. These symptoms contribute to foot ulcers and lower-limb amputations, leading to substantial physical and economic burden [4,5]. The estimated public health costs for DPN exceed $10 billion annually [6]. Developing effective treatments to address the prevalence and societal impact of DPN is necessary to provide relief from these symptoms.

**Figure 1:**
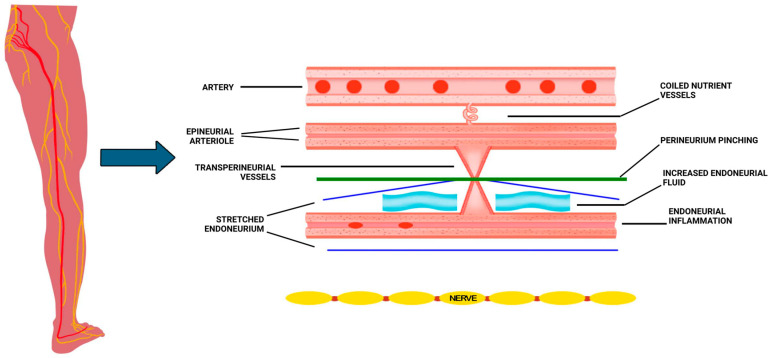
Neuropathy Neurovascular Circulation Schema.

Pathophysiology in DPN includes impaired neural circulation and disruption of neurovascular coupling drive axonal ischemia, demyelination, and progressive sensory loss [1]. Endothelial dysfunction reduces vasa nervorum blood flow, leading to endoneurial hypoxia and oxidative stress. Both experimental and clinical data demonstrate that micro-angiopathy within the nerve precedes overt neuropathy and parallels symptom severity [4,7,8,9,10]. These vascular changes compromise barrier integrity, increase fascicular pressure, and create a localized micro-compartment syndrome that perpetuates ischemia (Figure 1) [4,11,12,13,14].

Therapeutic approaches that restore microvascular function are therefore of major clinical interest. Exercise remains a cornerstone of diabetes management and has shown benefit in reducing pain and improving sensory function in DPN [11,15,16,17,18]. Exercise training has been shown to improve nerve conduction, endothelial nitric-oxide bioavailability, and microvascular perfusion in adults with DPN, leading to reduced neuropathic pain and improved sensory function [19]. Recent randomized trials confirm that structured aerobic, resistance, and balance-based programs can enhance functional outcomes and partially reverse small-fiber dysfunction in DPN [4,20,21]. However, many individuals with advanced neuropathic pain, impaired balance, or limited mobility are unable to tolerate moderate-to-vigorous exercise, creating a need for adjunctive or alternative interventions that can restore microvascular health without requiring sustained physical exertion [21].

Intraneural Facilitation^TM^ (INF^®^) therapy is a manual therapy technique developed to address these limitations. INF^®^ therapy uses biomechanical positioning holds intended to reduce vascular compression and facilitate reperfusion across the epineurial, perineurial, and endoneurial layers of the nerve [22,23,24]. By restoring flow from macrocirculation to endoneurial microvasculature, INF^®^ therapy aims to relieve ischemia and normalize intrafascicular pressure. Preliminary clinical studies at the Neuropathic Therapy Center (NTC) have shown reductions in pain severity and improvement in protective sensation following INF^®^ therapy [22,23,24].

While exercise remains an essential element of diabetes management, INF^®^ therapy may offer broader within-group benefits among individuals unable to participate in traditional training. Thus, comparative evaluation of these two approaches, active exercise versus passive microvascular restoration, is warranted to better define their respective contributions to pain modulation in DPN.

Beyond exercise-based rehabilitation and the present intervention, guideline-supported options for painful DPN include first-line pharmacologic agents such as serotonin–norepinephrine reuptake inhibitors, gabapentinoids, tricyclic antidepressants, and sodium-channel blockers [25]. Topical capsaicin patches (8%) are recommended for focal foot pain, and, in refractory cases, high-frequency (10-kHz) spinal cord stimulation has demonstrated sustained benefit [25]. Current guidelines emphasize minimizing chronic opioid use; although tapentadol is FDA-approved for painful DPN, it is generally not preferred for long-term management [25].

Despite promising results, comparative data between passive vascular-restoration therapy and conventional exercise remain limited. To address this gap, we hypothesized that both interventions would improve pain quality, with INF^®^ therapy showing broader within-group effects due to its targeted microvascular focus. To test our hypothesis, we performed a randomized controlled trial, conducted at the NTC in Loma Linda University Health, Loma Linda, California, that was designed to evaluate and compare the effects of INF^®^ therapy and a standardized physical-therapy exercise program on neuropathic pain quality as measured by the Pain Quality Assessment Scale (PQAS).

## 2. Materials and Methods

### 2.1. Study Design and Setting

This study followed a single-blind, parallel-group randomized controlled trial design conducted at the Neuropathic Therapy Center (NTC), Loma Linda University Health, Loma Linda, California, USA, between February 2023 and January 2025. The trial adhered to the CONSORT 2010 guidelines for randomized clinical trials. Ethical approval was obtained from the Loma Linda University Institutional Review Board (IRB #5220363), and the study was registered on ClinicalTrials.gov (NCT05577390) prior to enrollment. No changes to the trial design or study procedures were made after trial commencement. The full study protocol is publicly available on ClinicalTrials.gov.

### 2.2. Participants

Adults aged 45–85 years with T2D and clinically confirmed moderate to severe DPN were eligible for inclusion. Inclusion criteria were: (1) ≥neuropathic symptoms (numbness, tingling, burning, sharp pain, or increased sensitivity in the lower extremities); (2) ability to ambulate independently for ≥15 min; and (3) availability for nine treatment sessions over six weeks. Additional requirements included English fluency and access to a smartphone for data entry. Exclusion criteria included: non-diabetic neuropathies (e.g., vitamin B12 deficiency, Charcot-Marie-Tooth disease), active infection or ulcer, recent amputation, uncontrolled systemic disease, morbid obesity (body mass index [BMI] ≥ 40 kg/m^2^), pregnancy, smoking or marijuana use, poorly controlled clotting disorder, recent cardiac surgery, inability to maintain a steady finger for app-based testing, cardiac pacemaker, or metal allergy (cobalt, chrome, nickel). Individuals undergoing chemotherapy or radiation within six months were also excluded. Eligible participants provided written informed consent before study procedure commenced. A total of 38 participants met eligibility criteria and enrolled in the study (53% female, 47% male). Baseline demographic and clinical characteristics were comparable between groups prior to randomization.

### 2.3. Randomization and Blinding

Participants were randomly assigned (1:1 ratio) to either the INF^®^ therapy group or the standardized exercise group using computer-generated block randomization to ensure balanced allocation. Allocation concealment was maintained through sequentially numbered sealed envelopes prepared by an independent coordinator. Due to the nature of the interventions, therapists could not be blinded; however, outcome assessors administering the PQAS and data analysts remained blinded to treatment assignment throughout data collection and analysis. The randomization sequence was generated automatically through the study computer system at the time of enrollment. Participants were enrolled by study clinicians, and group assignments were revealed through the electronic allocation system. Outcome assessors remained blinded to all assignments.

### 2.4. Interventions

#### 2.4.1. Intraneural Facilitation^TM^ (INF^®^) Therapy

**Figure 2:**
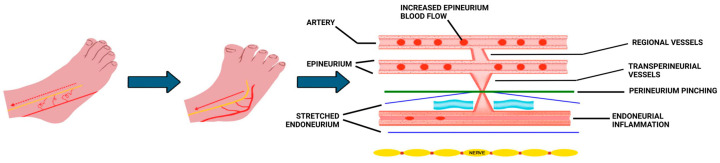
Facilitation Hold Schema.

**Figure 3:**
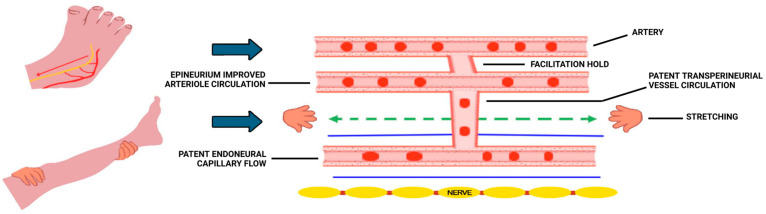
Secondary Hold Schema.

Participants in the INF^®^ therapy group received nine 60-min sessions delivered twice weekly by licensed physical therapists trained and certified in INF^®^ therapy. INF^®^ therapy is a manual technique designed to restore perfusion to ischemic nerves by biomechanically positioning the extremities to relieve vascular compression and facilitate endoneurial reperfusion [22,23,24]. Each treatment includes three standardized “holds” designed to:

- reduce arterial-neural compression and facilitate epineurial inflow (Figure 2);
- redirect epineurial pressure into transperineurial channels to increase endoneurial flow (Figure 3); and
- A third sub-hold to sustain proximal perfusion velocity through gentle joint biasing to minimize stasis. All sessions followed a standardized clinical research protocol validated for the NTC research setting. Treatment fidelity was confirmed via checklists completed at each session.

#### 2.4.2. Standard Exercise Program

The Exercise group completed nine 60-min sessions under the supervision of licensed physical therapists.

- Aerobic gait training (15 min at self-selected pace; rate of perceived exertion monitoring on a 6–20 Borg scale);
- Strength training (3 sets × 15 repetitions of knee bends, toe rises, hip extensions, adduction, and abduction);
- Flexibility training (5 min each for calves and hamstrings); and
- Balance training (single-leg stance × 5 trials per leg).

These exercise activities were based on previously validated DPN rehabilitation protocols [26,27,28,29]. Compliance was monitored by session attendance logs.

### 2.5. Outcome Measures

The primary outcome was neuropathic pain quality assessed by the PQAS, a validated 20-item questionnaire capturing multiple sensory dimensions across three domains (Paroxysmal, Superficial, and Deep). Each item is scored from 0 (“no pain”) to 10 (“worst imaginable”), with subscale and total scores calculated according to published guidelines [30,31]. PQAS was administered at baseline (Visit 1) and post-treatment (Visit 11). For analysis, domain-level means (Paroxysmal, Superficial, Deep) were also computed by averaging the relevant items for each participant. These domain-level PQAS means were designated as secondary outcomes to characterize symptom-specific changes.

### 2.6. Sample Size and Power Analysis

Power analysis (G*Power v3.1) indicated that 20 participants per group would detect a 25% difference in PQAS change scores (effect size f = 0.25, α = 0.05, β = 0.80). To account for 15% attrition, the target sample was 40. No interim analyses or stopping rules were planned or implemented.

### 2.7. Statistical Analysis

Data were entered into a secure REDCap (Research Electronic Data Capture, Vanderbilt University, Nashville, TN, USA) version 27. All statistical analyses were conducted in IBM SPSS Statistics, Version 27 (IBM Corp., Armonk, NY, USA). Data normality was assessed with the Shapiro–Wilk test. Within-group differences were analyzed with paired-sample *t*-tests, and between-group comparisons were assessed with independent-sample *t*-tests. Effect sizes were calculated using Cohen’s d. In addition, a linear mixed-effects model was used to test the effects of group, time, and group × time interaction, adjusting for age and BMI. Results are reported as least-square means with 95% confidence intervals. Statistical significance was set at *p* < 0.05.

## 3. Results

**Figure 4:**
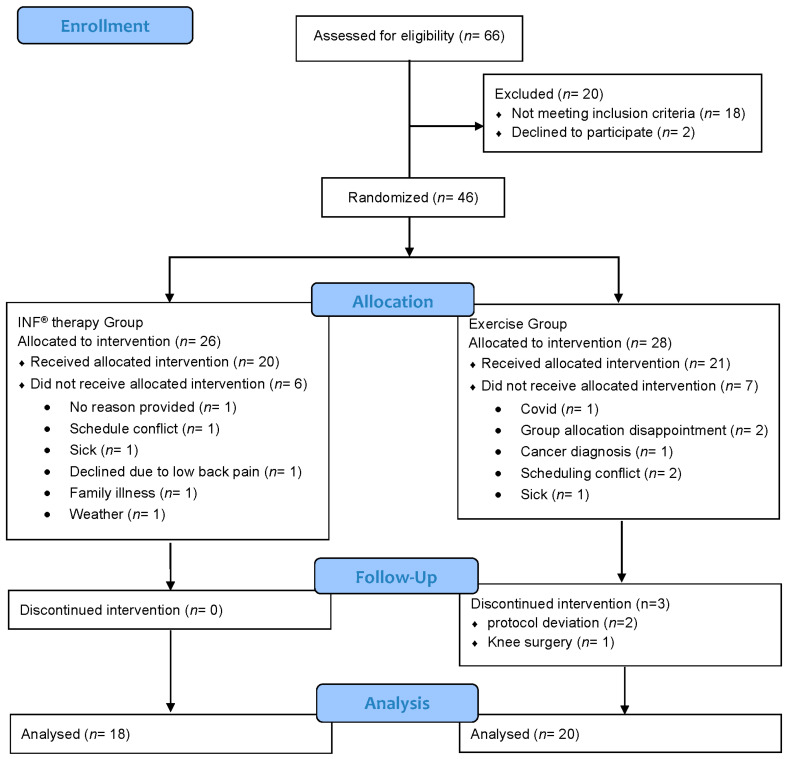
CONSORT flow diagram of participant recruitment, allocation, and follow-up.

The planned sample size was 40 participants aged 45–85 years with type II diabetic peripheral neuropathy. Recruitment occurred from February 2023 through November 2024, and all follow-up assessments were completed by January 2025. A total of 38 participants were enrolled and completed the study (INF^®^ therapy group, *n* = 20; Exercise group, *n* = 18) (Figure 4).

Baseline demographic and clinical characteristics were comparable between groups (Table 1).

Both groups demonstrated significant within-group reductions in PQAS total scores after six weeks (*p* = 0.001). Table 2 presents pre- and post-treatment scores for each PQAS item and domain.

In the INF^®^ therapy group, improvements were observed across all three domains. Paroxysmal symptoms improved, with reductions in shooting (*p* = 0.030), sharp (*p* = 0.001), electrical (*p* = 0.003), and radiating pain (*p* = 0.028). The paroxysmal domain mean also showed significant improvement (*p* = 0.002). Superficial symptoms decreased, including itchy (*p* < 0.001), cold (*p* = 0.030), numb (*p* = 0.002), and tingling (*p* < 0.001), with the superficial domain mean also improving (*p* < 0.001). For deep pain, we observed significant improvements in aching (*p* = 0.030), dull (*p* = 0.036), cramping (*p* = 0.037), and throbbing (*p* = 0.037), with the deep domain mean significantly reduced (*p* = 0.025). Global pain descriptors also improved, including intense (*p* = 0.017), unpleasant (*p* = 0.002), deep (*p* = 0.017), and surface pain (*p* = 0.020). Other descriptors, such as hot, heavy, and tender, did not reach statistical significance. The total PQAS score fell from 86.8 ± 40.7 to 51.9 ± 37.8 (*p* = 0.001, Cohen d = 0.83).

Within the exercise group, significant reductions were also observed, though in fewer domains (Table 2). Improvements were detected in sharp (*p* = 0.003) and electrical pain (*p* = 0.025), with corresponding improvement in the paroxysmal domain mean (*p* = 0.002). For superficial symptoms, numb (*p* = 0.029) and sensitive pain (*p* = 0.037) decreased, with the superficial domain mean also reduced (*p* = 0.002). Improvements were additionally observed in heavy (*p* = 0.024), intense (*p* = 0.014), unpleasant (*p* = 0.002), and surface pain (*p* = 0.025). No significant changes were observed for shooting, radiating, itchy, cold, tingling, aching, dull, cramping, throbbing, deep, or tender pain, nor for the deep domain mean. The total PQAS score decreased from 84.8 ± 38.1 to 58.4 ± 40.6 (*p* = 0.001, Cohen d = 0.99).

Independent *t*-tests and linear mixed-effects models revealed no significant group-by-time interactions for PQAS change scores (*p* > 0.05 for all items) (Table 2). After adjusting for age and BMI categories, the between-group differences remained non-significant, indicating both therapies were similarly effective in reducing neuropathic pain qualities over the six-week period. No protocol deviations were recorded.

All sessions were completed as scheduled under the supervision of certified therapists. Treatment fidelity checklists were completed for 100% of visits and showed excellent adherence to protocol. No unintended effects or adverse effects such as increased pain, fatigue, or muscle soreness were reported in either group.

## 4. Discussion

This single-blind randomized trial compared a novel passive manual therapy, INF^®^ therapy, with a standardized exercise program for adults with T2D and moderate-to-severe DPN. Both approaches significantly improved pain qualities measured by the PQAS. INF^®^ therapy produced broader within-group reductions across paroxysmal, superficial, and deep domains, whereas exercise improved selected descriptors. Between-group differences were not statistically significant after adjustment for age and BMI.

Exercise is an established intervention for DPN and has consistently reduced neuropathic pain [11,28]. Our findings confirm these benefits in a short-term, supervised setting. INF^®^ therapy, though less widely studied, has shown similar improvements in prior single-site studies [22,23,24]. The current trial reinforces those findings under randomized conditions, demonstrating safety, feasibility, and symptom relief comparable to active therapy.

Although both interventions reduce pain, the underlying mechanisms differ. Exercise likely acts through systemic pathways: increased endothelial nitric-oxide production, improved capillary recruitment, enhanced insulin sensitivity, and reduced pro-inflammatory cytokines [4,19,32]. INF^®^ therapy instead targets local neurovascular mechanics. By positioning joints to relieve perineurial compression, it may reopen transperineurial channels and restore endoneurial microcirculation. This concept aligns with prior imaging and microvascular studies showing that intraneural ischemia contributes to fascicular edema and pain [4,7,8,9,10,11,12,13,14]. Future investigations should incorporate objective physiological metrics such as near-infrared spectroscopy (NIRS) or laser Doppler flowmetry to confirm the hypothesized improvements in neural perfusion during and after INF^®^ therapy sessions. These measures would enable quantification of local vascular responses and help clarify whether perfusion restoration underlies observed pain reductions.

The practical advantages of each therapy differ. Exercise demands active participation and ongoing motivation but confers broad metabolic and cardiovascular benefits. INF^®^ therapy is passive and may be better suited for individuals who cannot safely exercise due to pain, weakness, or balance deficits. Combining both approaches could address systemic and local vascular dysfunction simultaneously, a strategy warranting investigation in larger multi-center trials. Additionally, multicenter trials should include long-term follow-up assessments extending beyond six weeks to determine the durability and sustainability of clinical benefits.

Moreover, future designs may benefit from including a sham-therapy or attention-control arm to account for non-specific therapeutic effects and the influence of therapist–patient interaction. Such control conditions would help isolate the specific physiological impact of INF^®^ therapy. Although full therapist blinding is inherently difficult in manual therapy studies, strategies such as standardized scripts, treatment fidelity monitoring, and cross-training across therapists may further minimize bias.

There are unanswered questions concerning both INF^®^ therapy, exercise and their relationship to the DPN pathophysiology involving the individual layers of the neural connective tissue. Exercise can be assumed to enhance macrocirculatory pressure and overcome DPN-associated arteriole and capillary resistance, partially accounting for the improvement in the researched metrics. However, the exact response of DPN neurovascular pathophysiology to exercise is unclear. Improved endothelial function [19], reduced insulin resistance [19], and decreased TNF-alpha levels [2] indicate systemic and probable improvement in neurovascular tissue. Post exercise nerve axon growth and increased nerve growth factor [19] indicate intrafascicular improvement with reduced endoneurial capillary dysfunction in DPN patients [1,20,33]. Still, the conspicuous absence of mechanistic data showing the impact of exercise on individual neurovascular layers on described pathophysiology of DPN patients leaves open the possibility of incomplete neurovascular healing despite systemic gains.

The concept of specifically targeting the individual layers of neural connective tissue to restore intrafascicular regulation of capillary flow and reduce endoneurial microcompartment syndrome with INF^®^ therapy is interesting yet there are significant gaps in our understanding of mechanisms. For instance, the force attenuation during the holds may not be sufficient to engage joint regional vessels. Also, the perineurium may not stretch sufficiently and with the necessary specificity to release “pinched” transperineurial vessels during INF^®^ therapy. However, the null hypothesis with this and other studies remain unsupported, and we recommend further work to develop animal models to test our hypotheses concerning INF^®^ therapy concepts.

The study’s modest sample size limited power to detect small between-group differences. All participants were treated at a single academic center, which may limit generalizability. Follow-up ended at six weeks, preventing evaluation of long-term durability. Although we adjusted for age and BMI, unmeasured confounding may persist. Objective physiologic data were not collected; integrating NIRS or flowmetry in future studies will strengthen mechanistic understanding of both INF^®^ therapy and exercise.

Both INF^®^ therapy and exercise produced significant short-term reductions in neuropathic pain qualities among adults with DPN. INF^®^ therapy showed broader within-group improvements, suggesting potential value as a passive adjunct or alternative when exercise participation is limited. These findings provide a foundation for larger mechanistic trials combining clinical and microvascular outcomes.

### Limitations

This study has several limitations. The small sample size and single-center design limit generalizability to broader populations. Replication across multiple sites is needed to confirm the external validity of these findings. The short six-week follow-up precluded assessment of long-term outcomes and durability of therapeutic effects. Despite blinding of assessors, therapist bias could not be completely eliminated, given that treatment providers were aware of group allocations. Objective physiological endpoints, such as NIRS or Doppler-based perfusion data, were not collected; integrating such measures in future studies will be essential to substantiate the hypothesized neurovascular mechanisms underlying INF^®^ therapy. While data integrity and statistical methods followed CONSORT guidelines, anonymized raw data and statistical code should be made available in future publications to enhance transparency and reproducibility. Finally, as Dr. Bussell is the inventor of INF^®^ therapy, the potential for investigator-related bias must be acknowledged, despite the study’s adherence to ethical and analytic standards.

## 5. Conclusions

Exercise and INF^®^ therapy are effective in reducing pain and paresthesia in patients with DPN as measured by the PQAS. Exercise remains an essential intervention due to its systemic benefits, while INF^®^ therapy offers a promising passive approach that may directly target microvascular dysfunction. By integrating findings from the present trial with prior INF^®^ therapy and exercise studies, this analysis highlights the potential for complementary strategies to address both systemic and localized contributors to neuropathic pain. Further investigation is warranted to determine the degree to which these approaches interact synergistically and to employ objective techniques such as near-infrared spectroscopy to assess their effects on neural microvascular function. The findings presented here provide a foundation for future investigations aimed at clarifying mechanistic pathways and optimizing integrative treatment strategies.

## Figures and Tables

**Table 1:** Demographic variables of subjects.

Variables	INF^®^ Therapy Group		Exercise Group	
Age	70.15 ± 7.08 ^a^		68.50 ± 4.05	
Height	1.70 ± 0.11		1.69 ± 0.09	
Weight	90.11 ± 23.17		82.73 ± 13.02	
BMI	30.97 ± 5.50		29.30 ± 5.35	
Gender	Female	9 (45.0) ^b^	Female	7 (38.9)
	Male	11 (55.0)	Male	11 (61.1)
Race	White or Caucasian	12 (60.0)	White or Caucasian	11 (61.1)
	Black or African American	2 (10.0)	Black or African American	3 (16.7)
	Asian	1 (5.0)	Asian	2 (11.1)
	Hispanic or Latino	2 (10.0)	Hispanic or Latino	2 (11.1)
	Two or more race groups	3 (15.0)	Two or more race groups	—

^a^: Mean ± SD. ^b^: count (percentage). Mean ± SD age was 69.3 ± 6.1 years, BMI 30.2 ± 5.4 kg/m^2^, and duration of diabetes 11.4 ± 3.8 years. Sex distribution was 53% female and 47% male. No adverse events occurred.

**Table 2:** Changes in Pain-Quality Assessment Scale (PQAS) Subscales and Domain Scores Following Intraneural Facilitation^TM^ therapy and Exercise in Adults with Diabetic Peripheral Neuropathy.

Questionnaire	INF^®^ Therapy Group *n* = 20				Exercise Group *n =* 18				

PQAS	Pre-Treatment	Post-Treatment	Cohen d	Statistical Analysis ^a^	Pre-Treatment	Post-Treatment	Cohen d	Statistical Analysis ^a^	Statistical Analysis ^b^
Shooting	3.65 ± 2.94 ^a^	2.15 ± 2.91	−0.53	*p =* 0.030	3.94 ± 3.80	2.94 ± 3.10	−0.37	NS	NS
Sharp	4.95 ± 3.05	2.35 ± 2.35	−0.85	*p =* 0.001	4.78 ± 3.64	2.78 ± 3.00	−0.80	*p* = 0.003	NS
Electrical	3.95 ± 2.95	1.95 ± 2.11	−0.75	*p =* 0.003	4.50 ± 3.59	2.83 ± 3.00	−0.58	*p* = 0.025	NS
Hot	2.60 ± 3.00	1.85 ± 2.58	−0.28	NS	4.00 ± 3.40	2.83 ± 3.19	−0.41	NS	NS
Radiating	3.60 ± 2.89	2.05 ± 2.52	−0.53	*p* = 0.028	2.72 ± 2.95	2.56 ± 2.66	−0.07	NS	NS
**Paroxysmal domain mean**	3.75 ± 2.40	2.78 ± 2.43	−0.78	*p* = 0.002	3.99 ± 2.55	2.79 ± 2.43	−0.86	*p* = 0.002	NS
Itchy	3.10 ± 2.59	0.80 ± 1.06	−0.94	*p* = 0.000	2.06 ± 3.24	2.06 ± 2.73	0.00	NS	NS
Cold	3.15 ± 3.48	1.70 ± 2.52	−0.52	*p* = 0.030	3.28 ± 3.41	2.17 ± 2.83	−0.35	NS	NS
Numb	5.70 ± 3.11	3.75 ± 3.08	−0.79	*p* = 0.002	5.67 ± 3.01	3.94 ± 2.86	−0.56	*p* = 0.029	NS
Sensitive	3.65 ± 3.72	2.50 ± 2.72	−0.39	NS	4.06 ± 3.56	2.17 ± 2.33	−0.53	*p* = 0.037	NS
Tingling	5.70 ± 2.79	2.90 ± 2.85	−0.94	*p* = 0.000	4.67 ± 3.05	3.50 ± 2.96	−0.36	NS	NS
**Superficial domain mean**	4.26 ± 1.84	2.33 ± 1.67	−1.16	*p* = 0.000	3.94 ± 1.94	2.77 ± 2.33	−0.85	*p* = 0.002	NS
Aching	4.15 ± 2.94	2.75 ± 2.83	−0.53	*p* = 0.030	3.67 ± 2.89	2.89 ± 2.65	−0.21	NS	NS
Heavy	4.25 ± 3.49	3.30 ± 3.16	−0.24	NS	3.56 ± 3.28	2.11 ± 2.61	−0.58	*p* = 0.024	NS
Dull	5.05 ± 2.87	3.15 ± 2.50	−0.50	*p* = 0.036	3.67 ± 2.57	3.61 ± 2.23	−0.02	NS	NS
Cramping	4.65 ± 3.36	3.25 ± 3.02	−0.50	*p* = 0.037	3.67 ± 3.27	2.33 ± 2.22	−0.39	NS	NS
Throbbing	3.15 ± 3.18	1.60 ± 2.33	−0.50	*p* = 0.037	3.39 ± 3.71	2.72 ± 2.95	−0.20	NS	NS
**Deep domain mean**	4.25 ± 2.57	2.81 ± 2.07	−0.54	*p* = 0.025	3.59 ± 2.23	2.73 ± 2.10	−0.44	NS	NS
Intense	5.35 ± 2.96	3.55 ± 2.76	−0.59	*p* = 0.017	5.67 ± 2.57	3.78 ± 2.76	−0.65	*p* = 0.014	NS
Unpleasant	6.30 ± 2.56	3.50 ± 2.76	−0.81	*p* = 0.002	7.06 ± 2.60	4.28 ± 3.06	−0.89	*p* = 0.002	NS
Deep	5.30 ± 3.13	3.55 ± 3.35	−0.59	*p* = 0.017	4.89 ± 3.58	3.61± 2.87	−0.33	NS	NS
Surface	5.10 ± 3.16	2.95 ± 2.63	−0.57	*p* = 0.020	5.06 ± 2.88	2.83 ± 2.62	−0.58	*p* = 0.025	NS
Tender	3.40 ± 3.00	2.25 ± 2.00	−0.37	NS	4.56 ± 3.71	2.44 ± 2.91	−0.53	*p* = 0.037	NS
Total Score	86.75 ± 40.68	51.85 ± 37.77	−0.83	*p* = 0.001	84.83 ± 38.12	58.39 ± 40.64	−0.99	*p* = 0.001	NS

Data are presented as mean ± SD. Statistical analysis ^a^ = within-group comparisons (paired *t*-tests with Cohen’s d effect size). Statistical analysis ^b^ = between-group comparisons (independent *t*-tests of change scores). *p* values < 0.05 were considered statistically significant. Results of between-group comparisons were additionally examined using mixed-effects models adjusted for age and body mass index (BMI) categories, which yielded consistent findings. INF^®^ therapy, Intraneural Facilitation^TM^ therapy; NS, not significant.

## Data Availability

De-identified data are available from the corresponding author upon reasonable request; public release is restricted to protect participant confidentiality in accordance with institutional and ethical guidelines.

## Abbreviations

The following abbreviations are used in this manuscript:

DPN	Diabetic peripheral neuropathy
T2D	Type 2 Diabetes
INF^®^ therapy	Intraneural Facilitation^TM^ therapy
NTC	Neuropathic Therapy Center
PQAS	Pain-quality assessment scale
REDCap	Research electronic data capture
BMI	Body mass index
NIRS	Near-Infrared Spectroscopy

## References

[1] Yang Y., Zhao B., Wang Y., Lan H., Liu X., Hu Y., Cao P. (2025). Diabetic neuropathy: Cutting-edge research and future directions. Signal Transduct. Target. Ther..

[2] Galiero R., Caturano A., Vetrano E., Beccia D., Brin C., Alfano M., Di Salvo J., Epifani R., Piacevole A., Tagliaferri G. (2023). Peripheral Neuropathy in Diabetes Mellitus: Pathogenetic Mechanisms and Diagnostic Options. Int. J. Mol. Sci..

[3] Chang M.C., Yang S. (2023). Diabetic peripheral neuropathy essentials: A narrative review. Ann. Palliat. Med..

[4] Jan Y.K., Kelhofer N., Tu T., Mansuri O., Onyemere K., Dave S., Pappu S. (2024). Diagnosis, Pathophysiology and Management of Microvascular Dysfunction in Diabetes Mellitus. Diagnostics.

[5] Zhu J., Hu Z., Luo Y., Liu Y., Luo W., Du X., Luo Z., Hu J., Peng S. (2023). Diabetic peripheral neuropathy: Pathogenetic mechanisms and treatment. Front. Endocrinol..

[6] Gordois A., Scuffham P., Shearer A., Oglesby A., Tobian J.A. (2003). The Health Care Costs of Diabetic Peripheral Neuropathy in the U.S. Diabetes Care.

[7] Schratzberger P., Walter D.H., Rittig K., Bahlmann F.H., Pola R., Curry C., Silver M., Krainin J.G., Weinberg D.H., Ropper A.H. (2001). Reversal of experimental diabetic neuropathy by VEGF gene transfer. J. Clin. Investig..

[8] Ward J.D. (1993). Abnormal microvasculature in diabetic neuropathy. Eye.

[9] Thrainsdottir S., Malik R.A., Dahlin L.B., Wiksell P., Eriksson K.F., Rosen I., Petersson J., Greene D.A., Sundkvist G. (2003). Endoneurial capillary abnormalities presage deterioration of glucose tolerance and accompany peripheral neuropathy in man. Diabetes.

[10] Mohseni S., Badii M., Kylhammar A., Thomsen N.O.B., Eriksson K.F., Malik R.A., Rosen I., Dahlin L.B. (2017). Longitudinal study of neuropathy, microangiopathy, and autophagy in sural nerve: Implications for diabetic neuropathy. Brain Behav..

[11] Gracia-Sanchez A., Lopez-Pineda A., Nouni-Garcia R., Zunica-Garcia S., Chicharro-Luna E., Gil-Guillen V.F. (2025). Impact of Exercise Training in Patients with Diabetic Peripheral Neuropathy: An Umbrella Review. Sports Med. Open.

[12] Grover-Johnson N.M., Baumann F.G., Imparato A.M., Kim G.E., Thomas P.K. (1981). Abnormal innervation of lower limb epineurial arterioles in human diabetes. Diabetologia.

[13] Teunissen L.L., Veldink J., Notermans N.C., Bleys R.L. (2002). Quantitative assessment of the innervation of epineurial arteries in the peripheral nerve by immunofluorescence: Differences between controls and patients with peripheral arterial disease. Acta Neuropathol..

[14] Beggs J., Johnson P.C., Olafsen A., Watkins C.J. (1992). Innervation of the vasa nervorum: Changes in human diabetics. J. Neuropathol. Exp. Neurol..

[15] Dubsky M., Veleba J., Sojakova D., Marhefkova N., Fejfarova V., Jude E.B. (2023). Endothelial Dysfunction in Diabetes Mellitus: New Insights. Int. J. Mol. Sci..

[16] Maiuolo J., Gliozzi M., Musolino V., Carresi C., Nucera S., Macri R., Scicchitano M., Bosco F., Scarano F., Ruga S. (2019). The Role of Endothelial Dysfunction in Peripheral Blood Nerve Barrier: Molecular Mechanisms and Pathophysiological Implications. Int. J. Mol. Sci..

[17] Gu D., Xia Y., Ding Z., Qian J., Gu X., Bai H., Jiang M., Yao D. (2024). Inflammation in the Peripheral Nervous System after Injury. Biomedicines.

[18] Sprenger-Svacina A., Svacina M.K.R., Otlu H.G., Gao T., Sheikh K.A., Zhang G. (2025). Endoneurial immune interplay in peripheral nerve repair: Insights and implications for future therapeutic interventions. Front. Neurosci..

[19] Luo J., Zhu H.Q., Gou B., Zheng Y.L. (2022). Mechanisms of exercise for diabetic neuropathic pain. Front. Aging Neurosci..

[20] Holmes C.J., Hastings M.K. (2021). The Application of Exercise Training for Diabetic Peripheral Neuropathy. J. Clin. Med..

[21] Akhtar S. (2024). Diabetes-induced peripheral neuropathy: Is prescribing physical exercise the answer?. Biomol. Biomed..

[22] Alshahrani A., Bussell M., Johnson E., Tsao B., Bahjri K. (2016). Effects of a Novel Therapeutic Intervention in Patients With Diabetic Peripheral Neuropathy. Arch. Phys. Med. Rehabil..

[23] Bussell M., Sahba K., Jahromi H., Rashidian M., Hankins J. (2025). A Retrospective Assessment of Neuropathic Pain in Response to Intraneural Facilitation^®^ Therapy and Neurovascular Index-Guided Food Elimination. Biomedicines.

[24] Sahba K., Berk L., Bussell M., Lohman E., Zamora F., Gharibvand L. (2022). Treating peripheral neuropathy in individuals with type 2 diabetes mellitus with intraneural facilitation: A single blind randomized control trial. J. Int. Med. Res..

[25] Price R., Smith D., Franklin G., Gronseth G., Pignone M., David W.S., Armon C., Perkins B.A., Bril V., Rae-Grant A. (2022). Oral and Topical Treatment of Painful Diabetic Polyneuropathy: Practice Guideline Update Summary: Report of the AAN Guideline Subcommittee. Neurology.

[26] Cruvinel-Junior R.H., Ferreira J., Verissimo J.L., Monteiro R.L., Silva E.Q., Suda E.Y., Sacco I.C.N. (2024). Affordable web-based foot-ankle exercise program proves effective for diabetic foot care in a randomized controlled trial with economic evaluation. Sci. Rep..

[27] Ferreira J., Cruvinel-Junior R.H., da Silva E.Q., Verissimo J.L., Monteiro R.L., Duarte M., Giacomozzi C., Sacco I.C.N. (2024). Effectiveness of a web-based foot-ankle exercise program for treating ulcer risk factors in diabetic neuropathy in a randomized controlled trial. Sci. Rep..

[28] Khurshid S., Saeed A., Kashif M., Nasreen A., Riaz H. (2025). Effects of multisystem exercises on balance, postural stability, mobility, walking speed, and pain in patients with diabetic peripheral neuropathy: A randomized controlled trial. BMC Neurosci..

[29] Nguyen V., Dinh Q., Yu F., Jia S., Wang X. (2025). Interventional effects of exercise on neuropathy in patients with diabetes: A systematic review with meta-analysis. BMC Sports Sci. Med. Rehabil..

[30] Jensen M.P., Gammaitoni A.R., Olaleye D.O., Oleka N., Nalamachu S.R., Galer B.S. (2006). The pain quality assessment scale: Assessment of pain quality in carpal tunnel syndrome. J. Pain..

[31] Victor T.W., Jensen M.P., Gammaitoni A.R., Gould E.M., White R.E., Galer B.S. (2008). The dimensions of pain quality: Factor analysis of the Pain Quality Assessment Scale. Clin. J. Pain..

[32] Hendrick E., Jamieson A., Chiesa S.T., Hughes A.D., Jones S. (2024). A short review of application of near-infrared spectroscopy (NIRS) for the assessment of microvascular post-occlusive reactive hyperaemia (PORH) in skeletal muscle. Front. Physiol..

[33] Yang J., Li L., Ye T., Pu Y., Yao Q., Luo J., Huang Y., Zhang X., Yang Z. (2025). Effectiveness of exercise on musculoskeletal function and clinical outcomes in patients with diabetic peripheral neuropathy: A systematic review and meta-analysis. Front. Neurol..

